# The influence of irrigation fluid temperature on recurrence in the evacuation of chronic subdural hematoma

**DOI:** 10.1007/s00701-019-04150-6

**Published:** 2019-12-04

**Authors:** Andreas Bartley, Asgeir S. Jakola, Magnus Tisell

**Affiliations:** 1 Department of Neurosurgery, Sahlgrenska University Hospital, Blå stråket 5, 41345 Gothenburg, Sweden; 2 Institute of Neuroscience and Physiology, Department of Clinical Neuroscience, University of Gothenburg, Sahlgrenska Academy, Box 430, 40530 Gothenburg, Sweden

**Keywords:** Chronic subdural hematoma, Irrigation fluid, Temperature, Surgical evacuation, Recurrence

## Abstract

**Background:**

Chronic subdural hematomas (cSDH) are one of the most common conditions requiring neurosurgical treatment. The reported recurrence after surgery is 3–21.5% with closed system drainage. In clinical practice, irrigation fluids at body temperature (37 °C) and at room temperature (22 °C) are routinely used in the evacuation of cSDH. Our hypothesis was that irrigation at body temperature might have more beneficial effects on coagulation and solubility of the chronic subdural hematoma than irrigation at room temperature. The aim of this study was to compare the effects of different intraoperative irrigation fluid temperatures on recurrence rates.

**Methods:**

This was a retrospective study where we included all consecutive patients from a defined geographical area of western Sweden between September 2013 and November 2014. In the course of 6 months, we performed intraoperative irrigation at body temperature (37 °C, BT-group) during burr hole evacuation of chronic subdural hematoma. This was then compared with the previous 6-month period, when irrigation fluid at room temperature (22 °C, RT-group) was used. The primary endpoint was same-sided recurrence in need of reoperation within 6 months.

**Results:**

Recurrence occurred in 11 of 84 (13.1%) patients in the RT-group compared with 4 of 88 (4.5%) in the BT-group (*p* = 0.013). There were no significant between-group differences regarding age, sex, duration of surgery, frequency of bilateral hematomas, hematoma density, and use of anticoagulant/antithrombotic therapy.

**Conclusion:**

Our study demonstrates that intraoperative irrigation fluid at body temperature is associated with lower recurrence rates compared with irrigation fluid at room temperature. To investigate this further, a prospective randomized controlled trial has been initiated (clinicaltrials.gov, NCT0275235).

**Trial registration:**

ClinicalTrials.gov Identifier: NCT0275235

## Introduction

Surgical evacuation of chronic subdural hematoma (cSDH) is one of the most common neurosurgical procedures. The incidence of cSDH is expected to increase due to the widespread use of anticoagulant/antithrombotic therapy together with an anticipated longer life expectancy in the general population [1, 2, 14]. Data from the Swedish National Board of Health show that the number of surgeries for cSDH has doubled in Sweden in the last 18 years [13].

The estimated recurrence of cSDH after surgical evacuation of cSDH is 3–21.5% with closed system drainage [16]. Reduction of recurrence rates may not only lessen the future load on the health care system but may also decrease the morbidity and mortality associated with recurrence [6, 10].

The most commonly used surgical procedure for cSDH treatment is evacuation via one or more cranial burr holes combined with postoperative drainage [8]. The procedure is often combined with perioperative irrigation of the subdural space. Although the benefits of perioperative irrigation have not been shown in a controlled study, there is evidence in favor of irrigation [7, 9].

A poll conducted on the neurosurgical networking site www.neurosurgic.com (accessed on 25th of September 2017) showed that out of 620 respondents, 57% used irrigation fluid at body temperature (37 °C), 40% used irrigation fluid at room temperature (22 °C), and 3% did not use irrigation at all. Thus, there was a large variation in practice concerning the temperature of the irrigation fluid. In theory, the temperature of the irrigation fluid might have an impact on recurrence, possibly due to improved coagulation and solubility of the cSDH when performing irrigation at body temperature versus irrigation at room temperature [4, 15]. However, no studies have evaluated the potential clinical impact of irrigation fluid temperature in the evacuation of cSDH.

The aim of this study was to explore whether or not the temperature of the irrigation fluid has an impact on recurrence rates. More specifically, we compared irrigation fluid at room temperature (RT-group) with irrigation fluid at body temperature (BT-group).

## Materials and methods

### Study design

Sahlgrenska University Hospital is the sole provider of neurosurgical care in western Sweden. The hospital has a well-defined geographical catchment area of about 1.8 million inhabitants. Approximately, 150–190 surgeries for cSDH are performed at our department annually. In the course of 6 months, between September 2013 and November 2014, we performed intraoperative irrigation at body temperature (37 °C) during burr hole evacuation of cSDH. Consecutive patients from this period were then compared with consecutive patients operated during the previous 6-month period when room temperature (22 °C) irrigation fluid was used. All patients had a postoperative follow-up of 6 months. The primary endpoint was recurrence requiring reoperation within 6 months. Secondary endpoints were complications requiring hospital admission and mortality.

### Surgical technique

The hematoma was evacuated via one or two burr holes combined with perioperative irrigation with Ringer’s lactate followed by active subgaleal drainage [5, 12]. The surgery was performed either under general or local anesthesia. The patient was kept in the supine position until the drain was removed the day after surgery. This surgical technique was adopted by all surgeons at our department.

### Postoperative management

Typically, most patients were sent to their local hospital the day after surgery. We do not routinely perform a postoperative CT scan at our department prior to discharge, except in the absence of improvement after surgery [11]. This policy was similar in both time periods. In total, 63% of the BT-group and 60% of the RT-group had at least one postoperative CT scan performed during the follow-up period. If the patient developed recurrent neurological symptoms (headache, hemiparesis, dysphasia, etc.), a CT scan at the local hospital was recommended and our department was contacted subsequently. We did not focus on minor morbidity during the postoperative follow-up but instead on mortality and morbidity requiring hospital admission. There was no change in postoperative management during the study period.

### Statistical analysis

The primary endpoint was analyzed with the *χ*^2^ test for frequency comparison. Secondary endpoints were analyzed with the *χ*^2^ test for categorical data. Normally distributed numerical data was analyzed with Student’s *t* test and the Mann-Whitney *U* test if skewed. Statistical significance was set at 5%.

### Ethical approval

The Regional Ethics Committee in Gothenburg, Sweden, reviewed this study and concluded that no formal approval was necessary due to its retrospective observational character.

## Results

Table 1 shows the baseline characteristics for the two groups. No statistically significant differences were seen between the groups regarding age, sex, anticoagulant/antithrombotic therapy, bilateral hematomas, hematoma density, maximum hematoma width, or duration of surgery.

**Table 1:** Characteristics of the compared groups. *BT* body temperature, *RT* room temperature

Variables	BT-group; *n* = 88	RT-group; *n* = 84	Difference
Mean age (SD)	74.5 (12)	75.1 (13)	*p* = 0.77
No. of females (%)	21 (24)	24 (28)	*p* = 0.27
Mean duration of surgery (SD)	44 min (20.6)	46 min (15.1)	*p* = 0.84
No. of patients treated with anticoag/antitromb (%)	40 (45)	40 (47)	*p* = 0.54
Mean maximal hematoma width (SD)	19 mm (7.1)	18 mm (6.7)	*p* = 0.34
CT image: hypodense (%)	38 (43)	35 (42)	*p* = 0.60
CT image: isodense (%)	25 (28)	19 (24)	*p* = 0.16
CT image: hyperdense (%)	10 (12)	11 (13)	*p* = 0.51
CT image: membranous (%)	15 (17)	18 (21)	*p* = 0.26
Bilateral hematoma (%)	18 (20)	15 (18)	*p* = 0.43

**Fig. 1:**
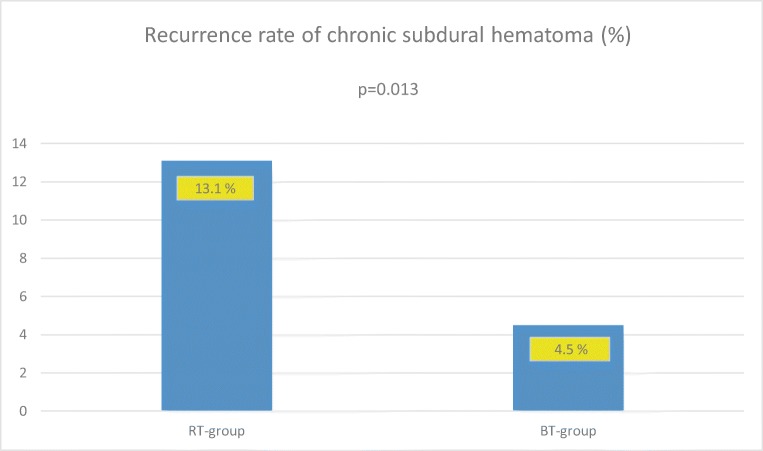
Frequency of recurrence between groups (*BT* body temperature, *RT* room temperature)

Recurrence within 6 months occurred in 11 of 84 (13.1%) in the RT-group compared with 4 of 88 (4.5%) in the BT-group (*p* = 0.013) (Fig. 1). All but one recurrence occurred within 2 months of surgery.

In total, three cases of mortality in the RT-group and four cases in the BT-group were noted during the postoperative follow-up period of 6 months. In the RT-group, one patient had a postoperative intracerebral hematoma and died within 30 days. In the BT-group, one patient suffered a basal ganglia infarction within 30 days, and another died of unknown cause at the local hospital 10 days after surgery. The remaining cases of mortality in both groups all occurred more than 2 months after surgery of causes unrelated to the surgery. If considering the postoperative hematoma and death of unknown cause as recurrences, the recurrence rates would be 14.2% for the RT-group and 5.6% for the BT-group, respectively (*p* = 0.016).

In addition to mortality, we also recorded complications leading to hospital admission during the follow-up period. In the BT-group, we recorded 1 case each of pneumonia, urinary tract infection, and non-ST elevation myocardial infarction (NSTEMI). In the RT-group we recorded 1 case each of wound infection and postoperative seizure. In addition, there were two cases (one in each group) of acute subdural hematomas requiring surgery. Furthermore, there were two cases of symptomatic pneumocephalus (one in each group), but neither required surgical intervention.

## Discussion

Our results show a statistically significant reduction in recurrence rates, from 13.1 to 4.5%, when using irrigation fluid at body temperature versus irrigation fluid at room temperature. There were no statistically significant differences in mortality or overall complication frequency between the two groups. To the best of our knowledge, this is the first study comparing the effects of different irrigation fluid temperatures used in the evacuation of cSDH.

Our finding indicates that the irrigation fluid temperature may affect recurrence rates. Theoretically, this might be explained by a negative effect on coagulation when using irrigation fluid at room temperature [15]. It is also possible that irrigation fluid at body temperature might increase the solubility of the cSDH, thereby facilitating evacuation [4].

Due to the expected increase in frequency of surgeries for cSDH, it is of great importance to try to optimize treatment strategies with evidence-based treatments [1, 2, 13, 14].

The strengths of this study are that the patients were included consecutively from a defined geographical area. Furthermore, digitalized medical records used at our department, as well as at the local hospitals in our population-based catchment area, enabled complete follow-up regarding mortality, recurrence, and hospital admissions. All cases of recurrence requiring surgery were treated at our department. Finally, one surgical technique was used when evacuating cSDH.

Limitations of the study include the retrospective design. Furthermore, the possibility of individual surgeons changing their surgical behavior when a study or a new intervention is undertaken cannot be overlooked, such as during the 6-month period when we changed our surgical routine to using irrigation fluid at body temperature. To address this matter, we used the duration of surgery as a surrogate marker for increased length of irrigation, which did not differ between groups.

## Conclusions

Our study shows that perioperative irrigation fluid at body temperature is associated with significantly lower recurrence rates compared with irrigation fluid at room temperature. In order to further investigate the results of this study, we have initiated a prospective randomized controlled trial (clinicaltrials.gov, NCT0275235) [3].

## Abbreviations

cSDH: Chronic subdural hematoma
BT: Body temperature
RT: Room temperature
